# Photoprotection of a novel resveratrol analogue: insights from 2D, 3D, and endothelialized bioprinted human skin models

**DOI:** 10.3389/fphar.2026.1756483

**Published:** 2026-04-22

**Authors:** Ana Júlia Pasuch Gluzezak, Lucie Essayan, Celine Thomann, Jean Leandro dos Santos, Christophe A. Marquette, Lorena Rigo Gaspar

**Affiliations:** 1 School of Pharmaceutical Sciences of Ribeirão Preto, University of São Paulo, Ribeirão Preto, São Paulo, Brazil; 2 3d.FAB, Université Lyon 1, CNRS, INSA, CPE-Lyon, ICBMS, Villeurbanne, France; 3 School of Pharmaceutical Sciences of UNESP, Araraquara, São Paulo, Brazil

**Keywords:** in vitro models, microvascularization, oxidative stress, photostability, tissue engineering

## Abstract

Excessive exposure to ultraviolet (UV) radiation induces oxidative stress, DNA damage, and extracellular matrix (ECM) disorganization, contributing to skin aging and disease. To enhance photoprotection strategies, antioxidant molecules have been explored as complementary agents to conventional UV filters. Here, we investigated the photoprotective potential of *N′,N‴,N‴'*'-((1*E*,1′*E,*1″*E*)-(((1,3,5-triazine-2,4,6-triyl)tris(oxy))tris(benzene-4,1-diyl))tris(methanylylidene))tris(2-hydroxybenzohydrazide) (Trz-HBH_3_), a novel resveratrol analogue obtained through molecular triplication. The compound was evaluated for UV absorption, photostability, phototoxicity in 3T3 fibroblasts, and protective effects against UVB-induced damage in HaCaT keratinocytes. To improve physiological relevance, three-dimensional (3D) human skin models were developed using fibroblasts and keratinocytes, with or without human umbilical vein endothelial cells (HUVEC), and assembled manually or by extrusion-based bioprinting. Models were characterized by Calcein-AM staining and (immuno)histochemical analysis. To assess oxidative stress responses, tissues were exposed to UVA radiation from two sources (UVA lamp and sun simulator), and intracellular reactive oxygen species (ROS) levels were quantified. While Trz-HBH_3_ showed no cytotoxic or phototoxic effects, it exhibited broad UV absorption (in the UVB, UVA II and part of UVA I), high photostability (remaining absorbance values of 93.8% ± 2.8% in the UVA region and 86.0% ± 8.0% in the UVB region), and protective effects against UVB-induced damage, maintaining cell viability close to 100%. Notably, Trz-HBH_3_ effectively reduced UVA-induced ROS by 63.5% and 69.5%, in both reconstructed and endothelialized bioprinted human skin models, respectively. These findings support the antioxidant and photoprotective properties of Trz-HBH_3_ and reinforce the applicability of bioprinted skin as an advanced *in vitro* platform to investigate UV-induced skin responses.

## Introduction

Despite the benefits of ultraviolet (UV) radiation, excessive exposure induces biological damages in the skin layers that contribute to photo-carcinogenesis and photoaging, due to cellular matrix disorganization and fibroblast alteration. Although the human body has natural protective mechanisms against UV radiation, such as thickening of the *stratum corneum* (hyperkeratosis) and skin pigmentation, these defenses become insufficient against environmental factors that make the skin vulnerable, necessitating additional protective measures (Stiefel and Schwack, 2015; Parrado et al., 2019).

It is well known that UV radiation can increase the generation of reactive oxygen species (ROS) in the skin, causing cellular function and extracellular matrix (ECM) alterations, DNA damage, along with a depletion of the endogenous antioxidant system, inflammation and immunosuppression (D’Orazio et al., 2013; Kochevar et al., 2012; Zamarrón et al., 2018; Wei et al., 2024). Therefore, the association between UV filters with high UV absorption and antioxidant compounds can offer enhanced protection against oxidative stress and inflammation, minimizing the cutaneous effects resulting from UV exposure. The development of preventive strategies against UV-induced damage is essential for maintaining skin health and requires that sunscreen formulations demonstrate both safety and efficacy (Stiefel and schwack, 2015). In this context, a new resveratrol-derived molecule has emerged as a promising candidate for multifunctional photoprotection. Due to its chemical structure, this compound exhibits strong UV-absorbing capacity, acting as a potential UV filter. In addition, its antioxidant activity contributes to the neutralization of ROS generated by UV radiation, supporting its role in the prevention of photoinduced oxidative stress (Polonini, 2013; Freitas et al., 2015; Freire et al., 2023; Gluzezak et al., 2024). The integration of such compounds into sunscreen formulations represents an innovative strategy to improve skin defense mechanisms against solar radiation.

Given the increasing UV exposure resulting from stratospheric ozone depletion, developing innovative and human-relevant *in vitro* models is crucial to better understand and mitigate photo-induced oxidative damage (Neale et al., 2025). In response to the need for physiologically relevant systems, three-dimensional (3D) engineered skin tissue have emerged as valuable tools, not only for clinical applications such as skin grafts, but also for studying skin disorders and evaluating the efficacy and safety of active compounds in cosmetics and pharmaceuticals. Over the past two decades, studies using monolayer cells (2D) and reconstructed human skin models (3D) have progressed significantly, providing a platform for evaluating the efficacy and toxicity of various compounds *in vitro* (Teimouri et al., 2018; Chunduri and Maddi, 2023). 3D skin models reproduce a structured epidermis and populated dermis, enabling the assessment of UV-induced damage and elucidation of photoprotection pathways.

However, vascularization remains a key challenge for enhancing the functionality, viability and physiological relevance of these models. To address this, human umbilical vein endothelial cells (HUVEC) can be incorporated into the dermal layer, promoting a connected microvascular network (Devillard and Marquette, 2023). This advance not only improves the physiological accuracy of the models but also provides a robust platform for studying the effects of UV radiation on skin tissues. In academia, continuous efforts are made to enhance the physiological relevance and complexity of human skin equivalents, aiming to improve experimental throughput while advancing biological fidelity (Quílez et al., 2024).

Recent advancements have also led to more sophisticated skin models, incorporating elements like hair follicles and immune cells to more closely mimic human skin’s complexity *in vivo* (Nakamura et al., 2018; Catarino et al., 2023). Additionally, cell printing has emerged as a promising biofabrication technique due to its capacity to place living cells in precise spatial arrangements (Kim et al., 2018). Bioprinting technology allows for *in vitro* creation of complex tissues that replicate the 3D structure of cells, ECM, and biomaterials, opening new avenues for studying intricate biological processes and testing compounds. Although extrusion bioprinting is widely used (Boularaoui et al., 2020), comparisons with traditional pipetting methods for reconstructing skin models remain scarce in the literature (Bagatin et al., 2023).

To enhance the physiological relevance and complexity of 3D skin models, a bioprinted human skin construct was developed, consisting of an endothelialized dermal layer overlaid by a fully differentiated epidermis, using pneumatic extrusion-based bioprinting. This model was characterized and evaluated in terms of its response to UVA radiation, offering a promising *in vitro* platform to investigate UV-induced effects on human skin.

In this context, a new strategy involving molecular triplication of the antioxidant compound resveratrol was employed to increase the electronic conjugation and photoprotective activity of its derivative, *N′,N‴,N‴'*'-((1*E*,1′*E,*1″*E*)-(((1,3,5-triazine-2,4,6-triyl)tris(oxy))tris(benzene-4,1-diyl))tris(methanylylidene))tris(2-hydroxybenzohydrazide) (Trz-HBH_3_) (Reis et al., 2019). The photoprotective potential of Trz-HBH3 was assessed through an integrated approach, combining UV absorption assays, cytotoxicity evaluation, and intracellular ROS quantification in a range of *in vitro* models, including 2D cell cultures, reconstructed human skin, and endothelialized bioprinted skin. The use of more complex 3D models, particularly those incorporating endothelial cells, enabled the investigation of not only the compound’s photoprotective efficacy but also the tissue-level response to oxidative stress, thereby contributing to a more comprehensive understanding of UVA-induced damage in human skin.

## Results

The present work describes the evaluation of the photoprotective potential and phototoxicity of a novel resveratrol-derived compound (Trz-HBH_3_) using a combination of *in vitro* approaches. Initially, the UV absorption spectrum and photostability of Trz-HBH_3_ were characterized and compared to those of commercial UV filters. The protective effect of Trz-HBH_3_ against UVB-induced damage was then assessed in HaCaT monolayer cultures by measuring cell viability. Phototoxicity was evaluated following OECD Test Guideline 432, using a fibroblast monolayer model. To complement conventional assays, advanced skin models were developed, including an *in-house* reconstructed human skin model and a bioprinted endothelialized model incorporating HUVEC. These models were characterized for cell viability and morphology over a 16-day culture period. Finally, both models were employed to assess Trz-HBH_3_’s capacity to reduce UVA-induced intracellular ROS levels, enabling a comprehensive evaluation of its efficacy in physiologically relevant skin platforms.

### UV absorption and photostability

**FIGURE 1:**
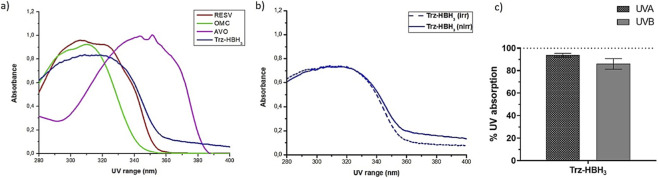
**(a)** Absorption spectra in the UV region (280–400 nm) of the UV filters avobenzone (AVO) and octyl methoxycinnamate (OMC), the antioxidant compound *t*-resveratrol (RESV) and Trz-HBH_3_ derivative (100 μg/mL) without UV irradiation; **(b)** Absorption spectra of the Trz-HBH_3_ derivative in solution before (nirr) and after (irr) UVA irradiation. Results are expressed as mean absorbance *(n* = 3); **(c)** Remaining UV absorption (%) in the UVA and UVB region for the Trz-HBH_3_ derivative. No significant differences were observed (t-test, *p* > 0.05). Statistical analysis was performed using Minitab (version 18), with *n* = 3 of independent experiments and a significance level set at *p* < 0.05. Figures were generated using GraphPad Prism (version 9.0) and OriginPro (8.5 version).

The UV absorption spectrum of the compound Trz-HBH_3_ (Figure 1a) revealed prominent absorption in the UVB range (280–320 nm), as well as in the UVA II (320–340 nm) and part of the UVA I (340–360 nm) regions, displaying a profile similar to that of resveratrol. Comparatively, the commercial UV filters avobenzone and octyl methoxycinnamate exhibited their characteristic absorption in the UVA (320–400 nm) and UVB (280–320 nm) regions, respectively. Notably, the absorption spectrum of Trz-HBH_3_ overlapped with that of octyl methoxycinnamate in the UVB region and partially with avobenzone in the UVA II/I range, suggesting potential for broad-spectrum photoprotection.

In terms of photostability, Trz-HBH_3_ solutions were exposed to UV radiation, and absorbance remaining was calculated by comparing the integrated areas under the absorption curve before and after irradiation. Non-irradiated samples were used as the baseline (100%). The Trz-HBH_3_ derivative demonstrated high photostability, with no significant changes in the absorption profile after irradiation (Figure 1b). Remaining absorbance values were 93.8% ± 2.8% in the UVA region and 86.0% ± 8.0% in the UVB region, indicating minimal photodegradation under the test conditions (Figure 1c). In contrast, previously obtained data for *t-*resveratrol showed a reduction in UV absorbance of 54.8% ± 12% in the UVA range and 53.2% ± 12% in the UVB range following irradiation (unpublished data).

### Photoprotective potential against UVB radiation

**FIGURE 2:**
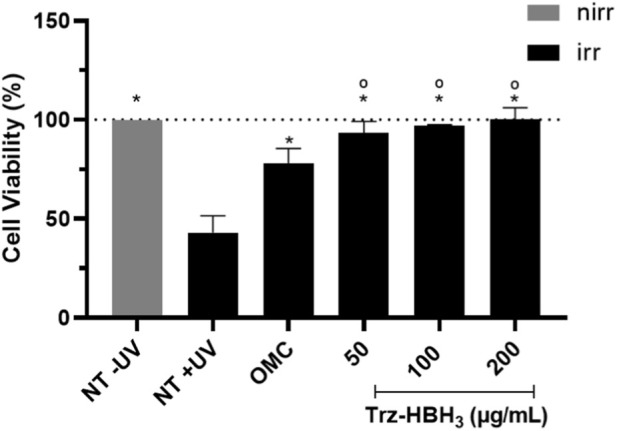
Cell viability of HaCaT keratinocytes following treatment with octyl methoxycinnamate (OMC) (100 μg/mL) and Trz-HBH_3_ derivative (50, 100, and 200 μg/mL), followed by UVB irradiation (300 mJ/cm^2^). NT -UV: untreated and non-irradiated control; NT + UV: untreated and irradiated control; OMC: octyl methoxycinnamate; Nirr: non-irradiated; Irr: irradiated. Results are expressed as mean ± S.E.M. (*n* = 3). *Statistically different from NT + UV (*p* < 0.05); °Statistically equivalent OMC (*p* > 0.05), according to one-way ANOVA followed by Tukey’s *post hoc* test. Statistical analysis was performed using Minitab (version 18), with *n* = 3 of independent experiments and a significance level set at *p* < 0.05. Figures were generated using GraphPad Prism (version 9.0).

The photoprotective potential against UVB radiation was assessed through a cell viability assay using HaCaT keratinocytes exposed to UVB. The viability of the untreated and irradiated control (NT + UV) decreased to 43% compared to the untreated and non-irradiated control (NT–UV) (*p* < 0.05) (Figure 2). The commercial UVB filter octyl methoxycinnamate, at 100 μg/mL, effectively protected HaCaT cells from UVB exposure, maintaining cell viability at 77.9% when compared to the NT + UV group (*p* < 0.05). Similarly, the Trz-HBH_3_ derivative, at all tested concentrations (50, 100, and 200 μg/mL), was effective in protecting HaCaT cells from UVB radiation, maintaining viability close to 100%. These values were statistically different from the NT + UV control (*p* < 0.05) and not significantly different from the octyl methoxycinnamate group (*p* > 0.05), suggesting a correlation between its absorption profile and photoprotective effects within the UVB range.

### Phototoxicity

**FIGURE 3:**
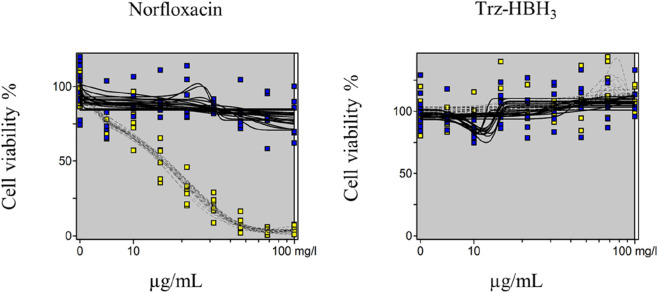
Dose-response curves of norfloxacin, used as positive control, and Trz-HBH_3_ derivative, obtained by the 3T3 NRU phototoxicity test and plotted using the Phototox Version 2.0 software. The blue and yellow dots refer to non-irradiated (nirr) and irradiated (irr) substances, respectively. Statistical analysis and curve fitting were performed using Phototox (version 2.0), with a significance level set at *p* < 0.05. The figure was generated using Phototox (version 2.0).

The phototoxicity results are presented in Table 1 and Figure 3. As expected, the positive control norfloxacin exhibited phototoxic potential, with Mean Photo Effect (MPE) values ranging from 0.34 to 0.9, in accordance with OECD Test Guideline 432 (OECD, 2019a). In contrast, the tested compound Trz-HBH_3_ showed no phototoxic potential, as it presented MPE values below 0.10 and did not exhibit cytotoxic effects on the cells.

**TABLE 1:** Results for 3T3 NRU phototoxicity assay for the samples: norfloxacin, positive control; Trz-HBH_3_ derivative. Results are expressed as mean photo effects (MPE) of two independent experiments.

Sample	MPE	Prediction model
Norfloxacin	0.568 0.520	Phototoxicity
Trz-HBH_3_	−0.035 −0.036	No phototoxicity

### Skin model characterization

**FIGURE 4:**
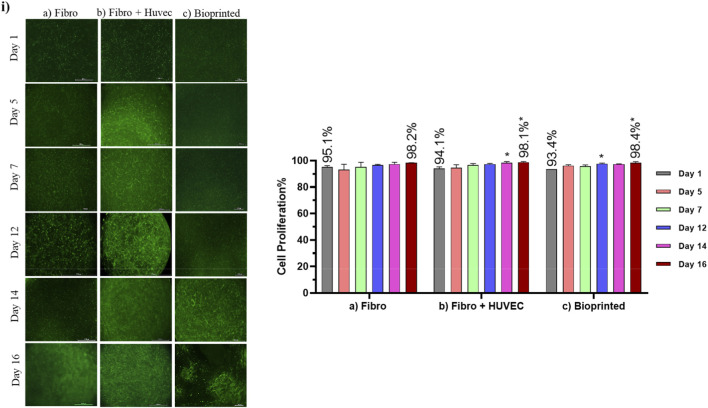
Fluorescence microscopy characterization of cell development in 3D collagen-based models over 16 days of *in vitro* culture. **(i)** Representative images of calcein-labeled cells in: **(a)** collagen I molded model with fibroblasts; **(b)** collagen I molded model with fibroblasts and HUVEC; and **(c)** bioprinted model with fibroblasts and HUVEC. Scale bar: 200 μm. ii) Quantitative analysis of cell proliferation (%) in the corresponding models over time. Data are expressed as mean ± SD (*n* = 3). Significantly different from day 1 (two-way ANOVA followed by Tukey’s test, *p* < 0.05). Statistical analysis was performed using Minitab (version 18), with *n* = 3 of independent experiments and a significance level set at *p* < 0.05. Figures were generated using GraphPad Prism (version 9.0) and microscope Leica (DM750, IBCP).

The collagen-molded models with only fibroblasts showed a homogeneous population of cells (Figure 4a), with cell density rising from 95.1% (day 1) to 98.2% (day 16), but when compared to the models in co-culture with endothelial cells, it can be seen that by day 5, the co-culture of cells populated faster the matrix, with well-organized structures (Figure 4b), suggesting that the presence of endothelial cells helps with fibroblast growth. The collagen-molded models with a co-culture of fibroblasts and HUVEC showed a significant increase in cell density from 94.1% on day 1%–98.1% by day 16 (*p* < 0.05) (Figure 4b). By day 5, the cells had populated the collagen matrix more rapidly, maintaining a visually homogeneous distribution. By day 7, the matrix displayed a dense cell network, suggesting a strong interaction between them.

The endothelialized bioprinted dermis, obtained by extrusion bioprinting of a cellularized hydrogel solution of gelatin, alginate and fibrinogen, showed a significant increase in cell density from day 1 to day 16, rising from 93.4% to 98.4% (*p* < 0.05) (Figure 4c). Early on, cells appeared sparsely distributed, but by day 14, proliferation became visually evident, aided by the bio-inspired bioink, which mimics the composition and mechanical properties of soft tissue, creating niches for tissue development. By the end of the culture, complex and well-organized structures were observed, indicating mature tissue formation.

**FIGURE 5:**
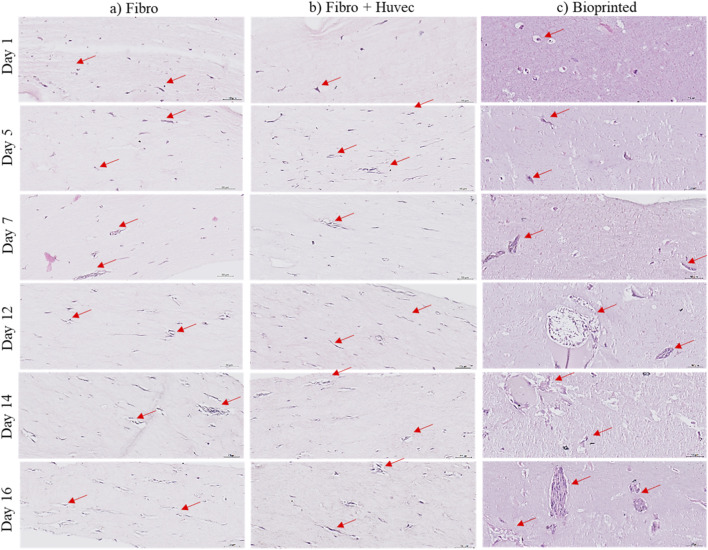
Histological characterization of 3D hydrogel or collagen I molded models stained with Hematoxylin and Eosin (HE), during 16 days of *in vitro* culture, according to three different models: **(a)** collagen I molded model with fibroblasts; **(b)** collagen I molded model with fibroblasts and HUVEC; **(c)** bioprinted model with fibroblasts and HUVEC. Scale bar: 50 µm. Figures were generated using microscope Leica (DM750, IBCP).

Hematoxylin and Eosin (HE) staining images (Figure 5) demonstrate that collagen-molded constructs containing only fibroblasts developed rapidly yet retained a relatively simple morphology throughout the 16-day culture period (Figure 5a). When endothelial cells were added to the collagen matrix, a marked improvement in cellular organization was already evident by day 5, pointing to enhanced extracellular-matrix deposition and the onset of microvascular assembly (Figure 5b). By contrast, the bioprinted hydrogels supported the formation of even more intricate three-dimensional structures: as early as day 7, fibroblasts and endothelial cells had self-organized into large interconnected microstructures indicative of angiogenesis, which further matured by day 16 (Figure 5c).

**FIGURE 6:**
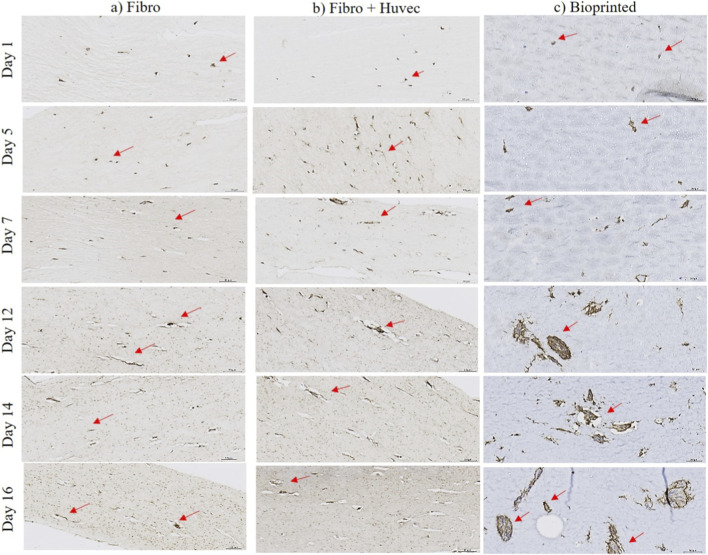
Immunohistological characterization of 3D hydrogel or collagen I molded models labeled with human collagen type I during 16 days of *in vitro* culture, according to three different models: **(a)** collagen I molded model with fibroblasts; **(b)** collagen I molded model with fibroblasts and HUVEC; **(c)** bioprinted model with fibroblasts and HUVEC. Red arrows represent staining of collagen I synthesis in the matrix. Scale bar: 50 µm. Figures were generated using microscope Leica (DM750, IBCP).

Type I human collagen immunolabeling confirmed ECM synthesis by fibroblasts in all tissue models. In collagen I molded constructs containing only fibroblasts, collagen deposition became more pronounced after day 7, indicating progressive ECM development (Figure 6a). When HUVECs were included, collagen neo-synthesis was enhanced, with strong brown signals already visible by day 5 and broadly distributed throughout the matrix (Figure 6b). In the bioprinted tissue, dense collagen staining was evident across the construct by day 16, with peak intensity observed between days 7 and 12 (Figure 6c).

**FIGURE 7:**
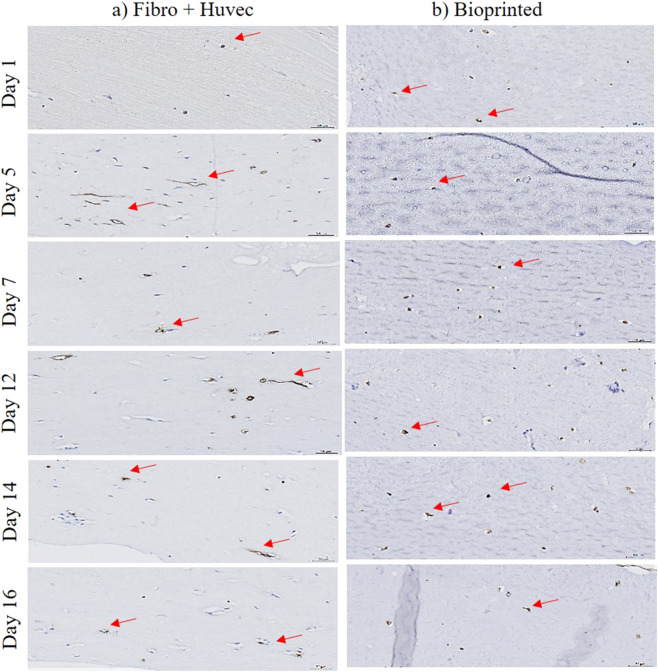
Immunohistological characterization of 3D hydrogel or collagen I molded models labeled with EN4 during 16 days of *in vitro* culture, according to three different models: **(a)** collagen I molded model with fibroblasts and HUVEC; **(b)** bioprinted model with fibroblasts and HUVEC. Red arrow represents EN4 staining of the endothelial cells in the models. Scale bar: 50 µm. Figures were generated using microscope Leica (DM750, IBCP).

The presence of HUVEC was visualized through EN4 immunohistological labeling, which binds specifically to endothelial markers on cell membranes (brown staining indicated by red arrows in Figure 7). EN4 stains endothelial cells at various stages, with stronger staining typically observed in mature cells due to increased marker expression. In the collagen I molded models (Figure 7a), circular structures formed by HUVEC were observed as early as days 5, 7, and 12, with subtle morphological changes emerging from day 5 onward. In contrast, the bioprinted model (Figure 7b) showed a more gradual development, with HUVEC evenly distributed throughout the matrix and self-organizing into small circular structures, which remained morphologically consistent over time. Despite the slower progression, the vascular organization in the bioprinted tissue may lead to a more stable and homogeneous microvascular network due to the precise spatial arrangement of HUVEC within the 3D matrix.

**FIGURE 8:**
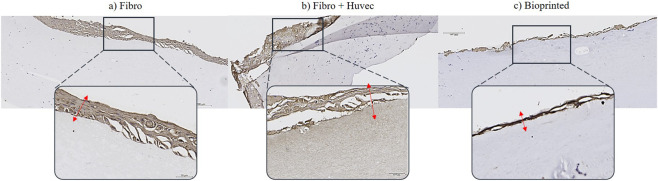
Immunohistological characterization of 3D hydrogel or collagen I molded models labeled with pan-cytokeratin, according to three different models: **(a)** collagen I molded model with fibroblasts; **(b)** collagen I molded model with fibroblasts and HUVEC; **(c)** bioprinted model with fibroblasts and HUVEC. Red arrow represents epithelium layer staining with pan-cytokeratin. Scale bar: 200 and 50 µm. Figures were generated using microscope Leica (DM750, IBCP).

Cytokeratin labeling confirmed the presence of an epidermal layer across all three models (Figure 8), using a mix of pan-cytokeratin to detect various stages of keratinocyte differentiation. In the collagen I molded models and those containing HUVEC, cytokeratin expression was consistently observed, indicating proper epidermal formation. Similarly, the bioprinted model exhibited uniform labeling, reflecting successful keratinocyte development and maturation. The consistent expression across models supports the effective establishment of an epidermal layer regardless of fabrication method.

### UVA-induced intracellular ROS production

After the first step of tissue characterization, skin models were subjected to an UVA exposure (10 J/cm^2^), emitted by a sun simulator with a peak in the UVA range or an UV lamp narrowly focused at 366 nm. The aim was to induce ROS generation and assess the photoprotective effect of the resveratrol’s analogue (compound Trz-HBH_3_) against UVA-induced intracellular ROS production, as well as the response levels from each developed model, using the DCFH_2_-DA probe.

**FIGURE 9:**
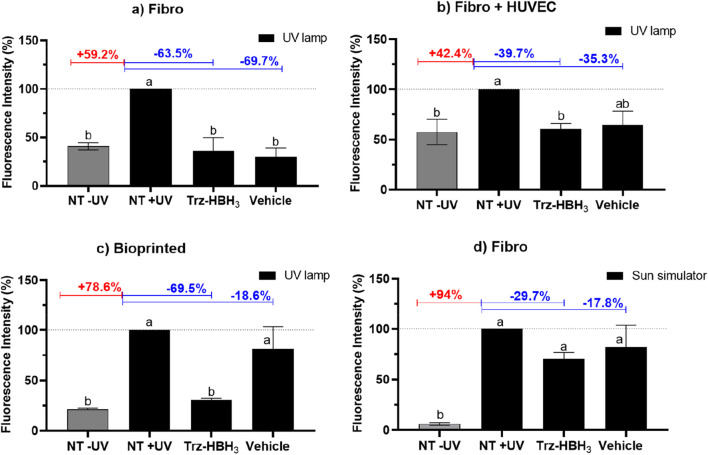
UVA-induced intracellular ROS production in the models: **(a)** Fibro: reconstructed human skin model (without HUVEC); **(b)** Fibro + HUVEC: reconstructed human skin model containing HUVEC; **(c)** Bioprinted: endothelialized bioprinted human skin model; **(d)** Fibro: reconstructed human skin model (without HUVEC) exposed to the sun simulator. Results are expressed as the percentage of fluorescence in comparison to the NT + UV. Untreated non-irradiated control (NT -UV); Untreated irradiated control (NT + UV); Trz-HBH_3_: resveratrol analogue obtained from a molecular triplication process (200 μg/mL); Vehicle: sesame oil; UV lamp: UVA-emitting lamp. Results are expressed as mean ± standard error of the mean from three independent experiments. Different letters indicate statistically different means (*p* < 0.05), according to one-way ANOVA followed by Tukey’s *post hoc* test. Statistical analysis was performed using Minitab (version 18), with *n* = 3 of independent experiments and a significance level set at *p* < 0.05. Figures were generated using GraphPad Prism (version 9.0).

**FIGURE 10:**
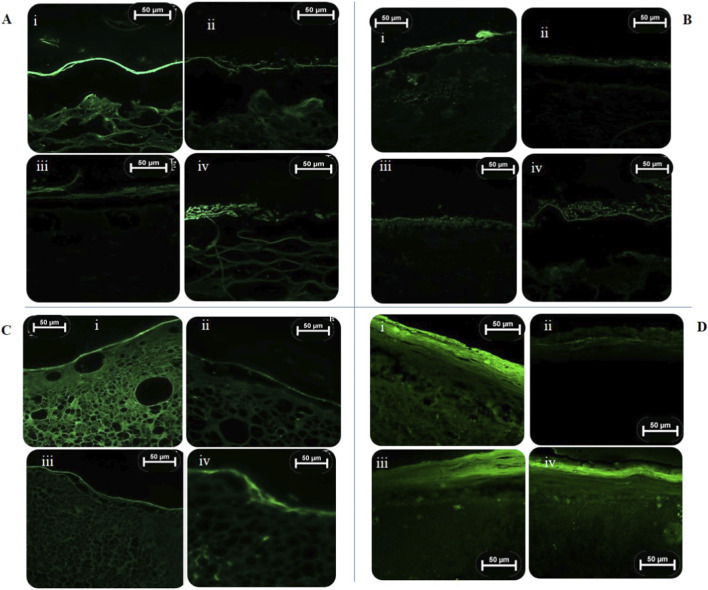
Fluorescence images obtained with confocal microscopy for each human skin model: **(A)** collagen I molded model with fibroblasts; **(B)** collagen I molded model with fibroblasts and HUVEC; **(C)** bioprinted model with fibroblasts and HUVEC; **(D)** collagen I molded model with fibroblasts exposed to the sun simulator. (i) Untreated irradiated tissue (NT +UV); (ii) Untreated non-irradiated tissue (NT -UV); (iii) Antioxidant compound Trz-HBH_3_ (resveratrol analogue) (200 μg/mL); (iv) vehicle, sesame oil. Green fluorescence corresponds to the presence of ROS in the tissue. Scale bar 50 μm. Figures were generated using microscope Leica (DM750, IBCP).

As shown in Figures 9a, 10a, UVA radiation emitted by the UV lamp induced a significant increase of 59.2% in ROS generation in the reconstructed human skin model containing only fibroblasts when the NT -UV tissue is compared to the NT + UV tissue (*p* < 0.05). The compound Trz-HBH_3_ significantly protected this tissue, reducing ROS generation by 63.5% compared to the NT + UV group (*p* < 0.05), and the vehicle (sesame oil) also showed a significant reduction of 69.7% (*p* < 0.05). In the collagen I molded model containing HUVEC, UVA radiation also led to a significant 42.4% increase in ROS generation when comparing NT -UV and NT + UV tissues (*p* < 0.05) (Figures 9b, 10b). The compound Trz-HBH_3_ reduced ROS generation by 39.7% relative to the NT + UV tissue (*p* < 0.05). The vehicle led to a 35.3% reduction, presenting intermediate levels between the NT + UV and NT -UV groups (*p* > 0.05).

In the endothelialized bioprinted skin model, UVA radiation from the UV lamp induced a 78.6% increase in ROS generation when comparing the NT -UV to the NT + UV tissue (*p* < 0.05) (Figures 9c, 10c), indicating that this model is responsive to UV-induced stress through ROS production (fluorescence). The compound Trz-HBH_3_ significantly protected the bioprinted tissues, reducing ROS generation by 68.7% compared to NT + UV (*p* < 0.05), while the vehicle maintained ROS levels, with only an 18.6% reduction (*p* > 0.05).

Finally, the reconstructed skin model containing only fibroblasts was also exposed to a sun simulator. As shown in Figures 9d, 10d, UVA radiation from this light source induced the highest increase in ROS generation (94%) when the NT -UV tissue is compared to NT + UV (*p* < 0.05). Compared to the other skin models exposed to the UV lamp, the sun simulator resulted in a more intense ROS response, indicating stronger UV-induced stress. Compound Trz-HBH_3_ reduced ROS generation by 29.7%, while the vehicle led to only a 17.8% reduction. Neither treatment showed significant differences compared to NT + UV (*p* > 0.05).

## Discussion

Effective photoprotective formulations must prevent UV-induced molecular and cellular damage without compromising safety (Krutmann et al., 2020). Excessive UV exposure promotes oxidative stress, barrier disruption, and degenerative processes, reinforcing the need for photoprotective strategies that effectively protects against UV-induced stress (Ali et al., 2020; Larnac et al., 2024; Gluzezak et al., 2024; de Souza et al., 2025). Combining UV filters with antioxidants represents a rational strategy to enhance overall photoprotection (GASPAR and MAIA CAMPOS, 2006; Freitas et al., 2015).

Resveratrol, a polyphenol produced by plants in response to UV radiation, displays antioxidant, anti-inflammatory, immunomodulatory, and anticancer properties, particularly through free radical neutralization and inhibition of oxidative enzymes such as cyclooxygenase, lipoxygenase, and xanthine oxidase (Koushki et al., 2019; Biasutto et al., 2017; Lin et al., 2021; Gianchecchi and Fierabracci, 2020). Its analogues also demonstrate biological activities, and are under investigation for photoprotection due to their UV-absorbing capacity and potential to dissipate radiation as lower-energy molecules (Freire et al., 2021; Gluzezak et al., 2024). Incorporating these compounds into sunscreens is a promising strategy to enhance skin defense against UV-induced oxidative damage (Reis et al., 2014; 2019; Bhattacharya and Sherje, 2020).

To improve efficacy, molecular modifications have been applied to resveratrol to obtain derivatives with enhanced UV absorption, broader protection spectra, and increased stability (Lawrence et al., 2018; Reis et al., 2019; Gluzezak et al., 2024). In this context, we evaluated the resveratrol analogue Trz-HBH_3_ for its UV absorption profile, photostability, phototoxicity, and its protective effect against UVB-induced damage in HaCaT cells. In addition, we assessed its ability to reduce UVA-induced intracellular ROS in 3D models of reconstructed human skin and bioprinted endothelialized skin.

The absorption spectrum of derivative Trz-HBH_3_ shows strong absorption in the UVB, UVA II, and part of the UVA I regions, similar to its analogue resveratrol. This is due to its molecular structure containing aromatic rings, conjugated double bonds, and electron donor-acceptor groups, which promote electronic delocalization and molecular stabilization against solar photons (Shaath, 2010; Reis et al., 2014; 2019). Compared to common sunscreen filters, avobenzone (UVA) and ethylhexyl methoxycinnamate (UVB), both photounstable, Trz-HBH_3_ covers a broad UV spectrum, indicating potential as a broad-spectrum UV filter (Scarpin et al., 2021). Derivative Trz-HBH_3_ has a critical wavelength ≥370 nm, meeting the broad-spectrum UV protection criterion (Reis et al., 2019). This is attributed to hydroxyl groups *orto*-coupled to aromatic rings (Reis et al., 2019). Increasing conjugated double bonds and resonance structures enhances molecular stability and electronic conjugation (OSTERWALDER et al., 2014; Reis et al., 2014; Lawrence et al., 2018). Trz-HBH_3_ showed superior photostability, with low photodegradation and no significant difference between irradiated and non-irradiated samples, unlike avobenzone, which is prone to photodegradation and free radical formation upon UV exposure (Shaath, 2010; Gholap et al., 2023). Common stabilization strategies include combining avobenzone with other filters, photostabilizers, and antioxidants such as ubiquinone and *t*-resveratrol (GASPAR and MAIA CAMPOS, 2006; Afonso et al., 2014; Scarpin et al., 2021; Gluzezak et al., 2024). However, resveratrol’s antioxidant activity decreases after UV exposure due to *cis-trans* isomerization. These limitations justify the search for new derivatives like Trz-HBH_3_, which can prevent photodegradation, reduce free radical formation, and lower phototoxicity associated with UV exposure (Jordão et al., 2023).

The UVB photoprotective potential of derivative Trz-HBH_3_ was assessed by evaluating HaCaT keratinocyte viability after UVB exposure (de Souza et al., 2025). All tested concentrations provided protection, maintaining higher cell viability than the UV filter ethylhexyl methoxycinnamate, likely due to Trz-HBH_3_’s broad UV absorption spectrum and its molecular features, aromatic rings, conjugated double bonds, and electron-donating/withdrawing groups, that stabilize the molecule against solar photons (Reis et al., 2014; 2019; Gluzezak et al., 2024). Photoinstability of compounds compromises sunscreen safety and efficacy by generating reactive intermediates and reducing active molecules (GASPAR and MAIA CAMPOS, 2006). Photodegradation can produce ROS like superoxide anion (SA) and singlet oxygen (SO), key mediators of phototoxicity (Shaath, 2010). The photoreactivity assay (OECD TG 495) detects SO and SA upon UVA irradiation (OECD, 2019b). Previous studies on resveratrol derivatives indicate no photoreactivity, supporting their photoprotective potential (Gluzezak et al., 2024). In the phototoxicity assay (OECD TG 432), which measures 3T3 fibroblast viability after treatment and UVA exposure, Trz-HBH_3_ showed no phototoxic or cytotoxic effects (MPE <0.100), consistent with photo-stability and *in silico* predictions (OECD, 2019a). Despite possible false positives in 2D models due to lack of skin permeation assessment, this test remains the gold standard for acute phototoxicity evaluation (Ceridono et al., 2012; Tavares Spagolla Napoleão et al., 2020). Phototoxic substances should be further tested in 3D reconstructed skin models for confirmation (Liebsch et al., 2005; OECD, 2019a; 2023).

As an alternative to animal testing and for initial efficacy screening of new cosmetic ingredients, *in vitro* human skin cell cultures are widely used (Piasek et al., 2023). The demand for innovative and effective 3D human skin models has grown, supporting both clinical applications like skin grafts and fundamental pathophysiological research. These models reveal mechanisms of skin disorders and allow evaluation of active compounds’ efficacy and toxicity in cosmetic and pharmaceutical research (Salameh, 2021; Quílez et al., 2024). Advances in 3D bioprinting have enabled increasingly complex and physiologically relevant *in vitro* skin models. While 2D models were initially used for toxicity and efficacy studies, they lack the structural and functional fidelity of human skin (Quílez et al., 2024). The transition to 3D reconstructed skin models, developed manually or via bioprinting, has allowed incorporation of endothelialized dermis and mature epidermis, creating more representative platforms (Pontiggia et al., 2022). These 3D models enable physiologically relevant assessment of photoprotective and antioxidant compounds, as well as biological responses to UV radiation. Bioprinting provides precise control over cell and biomaterial organization, enhancing model complexity, biomimicry, and mechanical and biological functionality, improving their relevance for toxicological and efficacy assays (Jorgensen et al., 2020; Pontiggia et al., 2022). To improve the complexity of an *in-house* reconstructed human skin model and address gaps in the current literature comparing 3D skin model methodologies (Bagatin et al., 2023), we also developed and standardized an endothelialized human skin model using pneumatic extrusion-based bioprinting (Devillard and Marquette, 2023).

Initially, the models were characterized by fluorescence and (immuno)histochemical techniques, followed by analysis of intracellular ROS generation induced by UVA from two sources: UVA lamp and sun simulator. Over 16 days of *in vitro* culture, calcein fluorescence and HE staining showed that *in-house* reconstructed human skin models, created by pipetting fibroblasts into a type I collagen matrix, displayed a homogeneous cell population with active proliferation, development, and cellular stability, but lower structural complexity compared to co-cultures with endothelial cells. The addition of HUVEC endothelial cells to the collagen I skin model resulted in faster, more efficient, and homogeneous matrix colonization, suggesting endothelial cells promote fibroblast growth in the tissue (Belvedere et al., 2022). The endothelialized bioprinted model initially showed sparse cell distribution throughout the hydrogel, which self-organized into complex 3D microstructures over 16 days. This proliferation was supported by a bioinspired bioink (gelatin, alginate, and fibrinogen), mimicking the composition and mechanical properties of soft human tissues, creating favorable niches for tissue development (Marquette et al., 2024). In this context, gelatin was used to provide the hydrogel with suitable rheological properties during bioprinting and, being collagen-derived, supports cell adhesion and proliferation, promoting differentiation and tissue formation. Alginate maintained the 3D structure post-printing during incubation at 37 °C, due to crosslinking with a calcium ion (CaCl_2_)-based solution. Fibrinogen, a plasma-derived hydrogel, was added to enhance cell adhesion, as it forms a 3D matrix resembling a blood clot when combined with thrombin (Bociaga et al., 2019; Devillard and Marquette, 2023). This outcome can be attributed not only to the bio-inspired nature of the hydrogel but also to its unique rheological properties, including significant shear-thinning and a high static yield stress, providing an ideal environment for tissue formation (Marquette et al., 2024).

The inclusion of HUVEC in the dermis models appears to promote fibroblast proliferation, enhancing tissue organization. Compared to collagen I alone, the bioinspired hydrogel, designed to better replicate the *in vivo* environment, supported more structured tissue formation by the end of *in vitro* culture. While collagen I effectively supports cell growth, it may have limitations in mechanical and rheological properties, requiring concentration adjustments for optimal structural integrity (Montalbano et al., 2018; Mobaraki et al., 2020; Ellistasari et al., 2022). In contrast, the bioinspired hydrogel, which better mimics native tissue properties, facilitated the self-organization of HUVEC into 3D microstructures through fibroblast-HUVEC interactions (Devillard and Marquette, 2023), highlighting its potential in tissue engineering applications. HUVEC are the most commonly used endothelial cells in *in vitro* research (Wufuer, 2016; Mori et al., 2017), as they line blood vessels and play key roles in vascular development, nutrient transport, and response to mechanical stimuli (Cao, 2025). As highlighted by Devillard and Marquette (2023), the presence of fibroblasts during dermal bioprinting is essential for promoting angiogenesis and for ECM synthesis and maintenance. Vascular development relies on close interactions between fibroblasts, endothelial cells, and their surrounding microenvironment.

Immunostaining for human type I collagen confirmed ECM synthesis by fibroblasts in all three models analyzed. Collagen, as a major ECM component, is essential for initial cell adhesion and supports endothelial cell migration during vascular network formation (Minor and Coulombe, 2020). Fibroblasts in the collagen-based skin models were metabolically active, as evidenced by strong brown immunostaining. However, collagen neosynthesis visibly increased upon addition of HUVEC in both the collagen matrix and bioprinted models, suggesting that fibroblast–endothelial cell interactions enhance tissue development and functionality (Skrzypek et al., 2018; Shafiee et al., 2021). Fibroblasts can enhance microvessel formation, development and maturation, stabilizing endothelial cell-lined tube formation, and providing important signaling that leads to the maturation of capillaries made of HUVEC (Sukmana and Vermette, 2010). Previous studies indicate that co-culturing fibroblasts and HUVEC supports ECM production and angiogenesis, as FGF2 (Fibroblast Growth Factor 2) from fibroblasts promotes VEGF (Vascular Endothelial Growth Factor) expression in HUVEC, synergistically enhancing vascularization and tissue regeneration (Carmeliet and Jain, 2011; Fujita et al., 2023).

In the endothelialized bioprinted model, rounded brown collagen I staining likely indicates fibroblasts adopting a specific spatial arrangement, seemingly surrounding endothelial cells, producing ECM, and supporting vessel stabilization and angiogenesis. Previous DAPI and NG2 immunofluorescence labeling has shown that fibroblasts can adopt a pericytic phenotype, wrapping around HUVEC to stabilize emerging microvessels (Chae et al., 2023; Devillard and Marquette, 2023). Skin microvessels consist of endothelial cells supported by mural cells, primarily pericytes in microcirculation and smooth muscle cells in larger vessels. Pericytes are crucial for microvascular maturation and maintenance (Haase et al., 2019). Thus, pericytes or similar cells are often incorporated into *in vitro* models to replicate native microvasculature more effectively (Seymour et al., 2022). When endothelial cells form microvascular networks, stromal cells naturally associate to stabilize the new structures, a role fibroblast can fulfill by adopting a pericytic-like phenotype (Armulik et al., 2011; Stratman et al., 2009; Devillard and Marquette, 2023). Fibroblasts play a crucial role in ECM production, especially collagen I, which enhances tissue stability, supports microvessel maturation, and reinforces the ECM. ECM components and their remodeling are vital in regulating vasculogenesis and angiogenesis. Angiogenic factors, matrix-degrading proteases, and cell-ECM interactions influence stages of neovessel formation, including endothelial cell growth, migration and tube formation (Hosseini et al., 2022).

The presence of HUVEC was confirmed by immunohistochemical EN4 labeling, which binds specifically to endothelial cell membrane markers, with more intense staining typically observed in mature cells due to higher marker expression. In both endothelialized models, collagen-based and bioprinted, HUVEC were uniformly distributed throughout the matrix over 16 days of culture. Although vascular organization in the bioprinted model appeared to develop more gradually, it may lead to a more stable and homogeneous microvascular network due to the controlled spatial arrangement of HUVEC within the 3D matrix. In previous studies using the same hydrogel (fibrinogen, alginate, and gelatin) with co-cultured HUVEC and fibroblasts, Devillard and Marquette (2023) used confocal fluorescence microscopy with EN4 labeling to visualize the formation of a microvascular network after 7 days of culture. They clearly identified interconnected microvascular networks, indicating that branching morphogenesis had occurred within the hydrogel, a complex process rarely seen in *in vitro* vascular engineering (Salvucci et al., 2002; Chwalek et al., 2014). Additionally, electron microscopy revealed tight junctions and continuous basement membranes, which are essential for the development of microvascularization (Krüger-Genge et al., 2019). Given the intrinsic complexity of generating stable and mature microvascular networks in engineered tissues, achieving fully developed capillary structures typically requires fine-tuned temporal and biochemical cues and often iterative optimization of the model (Auger et al., 2013).

Finally, staining with a pan-cytokeratin mix confirmed the presence of the epidermal layer in all three models. The use of a pan-cytokeratin cocktail allowed detection of different stages of keratinocyte differentiation. The consistent expression across models indicated formation of the epidermal layer, reflecting keratinocyte development and maturation. Cytokeratins, intermediate filament proteins, are widely represented in human epithelial tissues with diverse molecular weights (40–68 kDa) and isoelectric points (pH 4.9–7.8). Different cytokeratin subtypes are characteristic of epithelial cell types and stages of differentiation, making them valuable in distinguishing epithelial malignancies and wound healing models (Moll et al., 1984; Jorgensen et al., 2020; Zhang et al., 2020; Poblete Jara et al., 2021). Proper epidermal stratification is essential to ensure skin barrier function and remains one of the most critical attributes of *in vitro* skin models, despite ongoing improvements (Mori et al., 2017; Quílez et al., 2024). Advances in 3D skin models require not only methodological innovation but also further studies to optimize and thoroughly characterize the resulting models to guarantee skin quality standards (Baltazar, 2020; Cubo et al., 2016; Salameh, 2021; Sriram, 2018; Quílez et al., 2024).

The characterization of the skin models demonstrated how matrix composition and HUVEC inclusion influence tissue organization and complexity. The bioinspired hydrogel provided a supportive environment for steady cell proliferation and enhanced structural organization, allowing for the formation of microvascular networks and stronger cell interactions. Meanwhile, the collagen I matrix supported vigorous cell growth with early cell clustering but achieved less structural complexity compared to the bioinspired hydrogel. The integration of HUVEC across both models promoted angiogenesis, improved collagen synthesis and contributed to the formation of complex 3D microstructures. Following this initial tissue characterization, the skin models were then subjected to UVA exposure to assess their response to external stressors, providing further insights into their potential for photoprotection studies and applications in regenerative medicine.

Initially, 3D skin models were exposed to UVA radiation (10 J/cm^2^) emitted either by a sun simulator with an UVA peak or an UV lamp at 366 nm. The aim was to induce intracellular ROS generation and assess the photoprotective effect of the resveratrol analogue (compound Trz-HBH3) against ROS production, as well as to compare the response levels of each developed model using the DCFH_2_-DA probe (Reis et al., 2014; Gluzezak et al., 2024). This probe is commonly used to evaluate intracellular ROS, such as hydroxyl radical, hydrogen peroxide, nitrite and carbonate anion. It undergoes hydrolysis by cellular esterases and, in the presence of these ROS, DCFH is converted into its fluorescent derivative DCF, which can be measured using various fluorescence-based techniques (Kalyanaraman et al., 2012; Murphy et al., 2022).

All 3D skin models exposed to UVA lamps showed significant differences in ROS generation between the control irradiated and non-irradiated groups, demonstrating their capacity to respond to this type of stress. The incorporation of HUVEC in the bioprinted and collagen-molded models adds an important dimension to the skin model by mimicking the microvascular environment found in *in vivo* skin. This vascular network could influence the distribution and effectiveness of topical compounds like the resveratrol analogue (Trz-HBH_3_). HUVEC may enhance the model’s responsiveness to oxidative stress induced by UVA radiation, making the models more physiologically relevant for assessing UV protection (Kwak et al., 2020; Sutterby et al., 2021). The presence of a vascular network in the models facilitates the evaluation of the interaction and migration of topically applied compounds, making them more relevant for formulation studies. However, it is important that the penetration of sunscreens into the deeper layers of the skin and their subsequent permeation be limited in order to minimize the risk of systemic toxicity (Freitas et al., 2015; Freire et al., 2023). The Trz-HBH_3_ derivative effectively protected the 3D skin models from UVA-induced ROS production. Incorporating antioxidants like resveratrol and its analogues into photoprotective formulations is a promising strategy to reduce UV-induced damage and free radical formation (Reis et al., 2014; 2019; Bhattacharya and Sherje, 2020). As ROS play a central role in initiating oxidative stress, inflammation, and ECM degradation, understanding their modulation under UVA exposure is essential for preventing photoaging and related skin disorders. The results in the 3D skin models support the photoprotective capacity of Trz-HBH_3_, highlighting its potential to prevent intracellular ROS generation triggered by UVA exposure.

Finally, the collagen I model with fibroblasts exposed to the sun simulator showed higher ROS generation than the models exposed to the UVA lamp, indicating a stronger response to UV-induced stress. Although compound Trz-HBH_3_ demonstrated photoprotective effects by reducing ROS generation in 3D reconstructed skin models, under the experimental conditions of this study, its efficacy was insufficient to significantly reduce ROS production induced by the sun simulator, which emits a broad spectrum of UVA radiation. Even with similar peaks at 366 nm and the use of UV lamps in the studies of photoinduced inflammation, ROS generation, photostability of sunscreens (Kumar et al., 1997; Swalwell et al., 2012; Pescia et al., 2012; Lewicka et al., 2013), the broader spectrum and potential higher intensity of the sun simulator likely result in higher ROS production. Studies show that the exposure to sun simulators can increase ROS, trigger immunosuppression and stimulate cytokine release (IL-1α, TNFα, MMP). The additional wavelengths emitted by the sun simulator may activate more photosensitizers and pathways leading to a more pronounced ROS generation (Sayre et al., 1990; Svobodová et al., 2007; Marrot et al., 2010; Sayre and Dowdy, 2010; Svobodová and Vostálová, 2010; Liebel et al., 2012; Onoue et al., 2014). This broader activation could explain the observed differences in ROS levels in the skin models between the two light sources.

Here we present new results regarding the exposure of endothelialized bioprinted human skin models to UVA radiation for intracellular ROS induction using the DCFH_2_-DA probe. Typically, in bioprinted models, UV radiation is applied for biomaterial photopolymerization or sterilization during bioprinting to prevent microbial contamination (Zennifer et al., 2022; Carranza et al., 2023; Huang et al., 2024). Previous studies have used the DCFH_2_-DA probe to assess the antioxidant potential of alginate hydrogels in cartilage tissues exposed to hydrogen peroxide (H_2_O_2_), simulating cellular oxidative stress (Genç et al., 2021; Zhang et al., 2023). Fayazbakhsh et al. (2023) developed a microfluidic chip with dynamic flow simulating blood vessels lined with HUVEC to investigate the antioxidant properties of resveratrol nanoparticles, confirming oxidative stress and endothelial dysfunction through increased ROS and multinucleated cells observation.

Overall, our findings highlight active cellular communication that supports matrix synthesis and tissue remodeling, contributing to the formation of a connected microvascular network within the hydrogel construct. Despite the physiological relevance of the experimental models used, certain study limitations should be acknowledged. As an *in vitro* investigation, the system does not fully reproduce the complexity of *in vivo* skin biology, including systemic immune responses, metabolic clearance mechanisms, and long-term exposure dynamics. Additionally, the low aqueous solubility of Trz-HBH_3_ may pose challenges for future formulation development and translational applications.

Nevertheless, the use of reconstructed and endothelialized bioprinted skin models provides a relevant platform to evaluate the photoprotective potential of novel sunscreen derivatives, offering valuable insights for the development of topical strategies against UV-induced damage. In a global context increasingly focused on phasing out animal testing, 3D human-based models are gaining greater importance. These advanced *in vitro* systems not only align with ethical and regulatory trends but also provide more physiologically relevant insights into human responses, reinforcing their role in next-generation safety and efficacy assessment (Department for Science, Innovation and Technology, 2025).

## Conclusion

Here, we conducted a comprehensive evaluation of the photoprotective potential and toxicity of the resveratrol-derived compound Trz-HBH_3_ using *in vitro* assays and advanced human skin models as alternatives to animal testing. The compound demonstrated broad-spectrum UV absorption, photostability, and no cytotoxic or phototoxic effects in fibroblasts. It effectively protected HaCaT cells from UVB-induced cytotoxicity and significantly reduced UVA-induced intracellular ROS generation in reconstructed and bioprinted endothelialized human skin models, underscoring its potential for inclusion in photoprotective formulations.

To better understand skin responses to UVA-induced oxidative stress, we developed and characterized *in vitro* human skin models containing or lacking endothelial cells in the dermis, produced either manually (by pipetting) or via pneumatic-extrusion bioprinting. These models addressed a relevant gap in the literature regarding how structural complexity and cell composition influence biological outcomes. The incorporation of HUVEC into the dermal compartment proved to be critical for mimicking the native microvascular environment, contributing to ECM production, cellular organization, and overall tissue viability.

Upon UVA exposure, we observed that factors such as matrix composition, presence of endothelial cells, fabrication method, and radiation source significantly impacted intracellular ROS production. Manually molded collagen I-based models exhibited distinct ROS profiles depending on HUVEC inclusion and exposure to compound Trz-HBH_3._ In bioprinted skin models, the ROS difference between irradiated and non-irradiated conditions was more pronounced, indicating greater sensitivity to UVA. Notably, the sun simulator induced a stronger oxidative response than the UVA lamp.

By comparing bioprinted and traditionally molded models, we demonstrated how increasing model complexity enhances physiological relevance and influences compound performance. These findings support the photoprotective potential of Trz-HBH_3_ and underscore the value of endothelialized bioprinted skin as a robust platform for evaluating UV-induced skin damage and screening candidate compounds. While further validation is needed to strengthen translational applicability, this study advances both the development of new photoprotective agents and the implementation of complex 3D models in scientific research.

## Methods

### Obtaining the resveratrol analogue derivative

**FIGURE 11:**
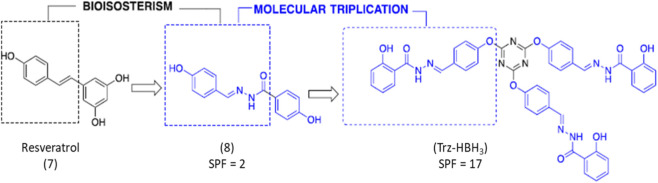
Molecular triplication process to obtain the Trz-HBH_3_ derivative according to Reis et al., 2019.

The compound under study was obtained based on the molecular triplication strategy described by Reis et al. (2019). In this approach, a central triazine core acts as the central scaffold, linked to three identical molecules containing the N-acyl-hydrazone subunit, an isostere of the double bond present in the precursor molecule, resveratrol (Figure 11). According to previous studies, this synthesis achieves a high yield of 65.4%. Following synthesis, the hydrazone derivative, designated as compound Trz-HBH_3_ (referred as 12C in the original manuscript), was characterized by mass spectrometry, infrared spectroscopy, and nuclear magnetic resonance (^1^H and ^13^C NMR). Additionally, molecular conjugation optimization was performed, yielding new resveratrol analogues, including compound Trz-HBH_3_, with a molecular weight of 843.8 g/mol. Further physicochemical properties of the compound are detailed in the supplementary material of Reis et al. (2019). According to MarvinSketch software predictions, compound Trz-HBH_3_ has a pKa of 7.55, a logP of 6.657, and low aqueous solubility. The pkCSM program indicates no predicted skin toxicity or sensitization, as well as low skin permeation values.

### Evaluation of photostability by UV spectrophotometry

To assess the photoprotective potential of Trz-HBH_3_, a photostability assay was conducted irradiating samples at a dose of 27,6 J/cm^2^ emitted by a UVA lamp (Philips UVA Actinic BL/10, Netherlands) (ICH, 1996; 1998), while the negative controls were kept in a dark place. For comparison, solutions of avobenzone, octyl methoxycinnamate and resveratrol were prepared at 100 μg/mL in isopropanol. The derivative was tested at 100 μg/mL in isopropanol. Solutions were analyzed by spectrophotometry in the 280–400 nm range. The results are expressed as a percentage of the area of irradiated samples related to the area of non-irradiated samples according to Gaspar and Maia Campos (2006), Tavares Spagolla Napoleão et al., 2020 and Gluzezak et al. (2024).

### Photoprotective potential against UVB radiation

The photoprotective potential of compound Trz-HBH_3_ against UVB-induced cytotoxicity was assessed by measuring cell viability following exposure to a cytotoxic dose of UVB radiation, as previously described (Lawrence et al., 2018). Human immortalized keratinocytes (HaCaT) were purchased from the Cell Bank of Rio de Janeiro (Rio de Janeiro, Brazil) and used up to 15 passages. 1 × 10^5^ cells/well were seeded in 96-well plates and incubated for 24 h at 37 °C in a humidified atmosphere with 5% CO_2_. Cells were then treated with Trz-HBH_3_ at concentrations of 50, 100, and 200 μg/mL. For comparison, cells were also treated with octyl methoxycinnamate (100 μg/mL), a commonly used UVB filter. After 1 h of treatment, plates were either kept in the dark or irradiated with UVB at a dose of 300 mJ/cm^2^ using a Philips Broadband UVB TL 40W/12 RS lamp. Non-treated irradiated cells (NT + UV) and non-treated non-irradiated cells (NT -UV) served as positive and negative controls, respectively. Following irradiation, cells were washed with PBS and incubated for an additional 24 h under standard culture conditions. Cell viability was determined using the neutral red uptake assay. Briefly, cells were incubated with neutral red diluted in culture medium, then washed and treated with a desorption solution (ethanol:water:acetic acid, 50:49:1, v/v/v). Absorbance was measured at 540 nm using a microplate reader. Results were expressed as a percentage of absorbance relative to the NT -UV control, considered as 100% cell viability.

### Phototoxicity assay (3T3 PT NRU)

The 3t3 Neutral red uptake phototoxicity (3t3 NRU PT) test was performed in accordance to OECD TG n° 432 (OECD, 2019a) using 3T3 BALB/c fibroblasts cells purchased from the Cell Bank of Rio de Janeiro (Rio de Janeiro, Brazil) and used up to 15 passages. Fibroblasts were cultured in DMEM supplemented with calf serum (10% v/v), L-glutamine (4 mM) and antibiotic mixture (penicillin, streptomycin and amphotericin B) and incubated at 37 °C with 5% CO_2_. The DMSO stock solution of Trz-HBH_3_ was at 100 μg/mL. These preparations were also diluted in PBS (pH 7.2) to generate samples with 8 different concentrations in a geometric progression (constant factor = 1.47). The highest final concentration of DMSO was 1%. Fibroblasts were seeded at a density of 1 × 10^4^ cell/well and after 24 h of incubation (5% CO_2_;37 °C), they were treated with 8 different concentrations of the combinations in the wells in sextuplicate, incubated for 1 h (5% CO_2_; 37 °C) and irradiated with UVA radiation of 9 J/cm^2^. This dose was selected according to the OECD 432 guideline (OECD, 2019a) for not being cytotoxic and sufficiently potent to excite norfloxacin to elicit phototoxic reactions. For each irradiated plate, there was a negative control (without UVA radiation) that was kept in the dark. After irradiation, the samples tested were replaced with culture medium and the plates were incubated for 18–22 h (5% CO_2_,37 °C). Cell viability was measured using the NRU assay with the uptake of the vital dye neutral red into cellular lysosomes. Cells were washed with PBS and incubated with culture medium containing 50 μg/mL of the neutral red vital dye for 3 h. Then a solution containing ethanol: water: acetic acid (50:49:1, v/v/v) was added to the plates. After measuring the absorbance of both plates at 540 nm using a microplate reader (SynergyTM 2, Biotek), all data were analyzed by Phototox Software 2.0 (ZEBET, Germany), and the mean photo effect (MPE) was calculated. According to the prediction model, a test substance with a MPE >0.15 is predicted to be “phototoxic,” a MPE >0.1 and <0.15 is predicted to be “equivocal phototoxic” and a MPE <0.1 is predicted to be “non-phototoxic” (OECD, 2019a).

### Reconstructed human skin model

*In-house* reconstructed human skin models were prepared in 24-well plates using primary human dermal fibroblasts and keratinocytes isolated from foreskin tissue (pooled from three donors aged 1–10 years). Fibroblasts and keratinocytes were used up to passage 7 and passage 5, respectively. The study was approved by the Ethics Committee for Research Involving Human Beings of the School of Pharmaceutical Sciences of Ribeirão Preto, University of São Paulo (CAAE n° 69358323.9.0000.5403; Protocol CEP/FCFRP n°551). The endothelial cells (HUVEC, Human Umbilical Vein Endothelial Cells, C-12203, PromoCell) were primary cells from a pool of 3 donors, used up to 7 passages. An informed consent was obtained from all subjects and/or their legal guardian(s). All methods were performed in accordance with the relevant guidelines and international and national regulations.

Primarily, the dermal compartment consisted of 3.43 mg/mL of collagen type I (354236, Corning) and 1.14 × 10^5^ normal human fibroblasts that were seeded into the insert (0.4 μm pore size; ThinCert™, Greiner Bio-One GmbH, Frickenhausen—Germany), with and without 1 × 10^6^ endothelial cells, and incubated overnight. After 20 h, 3.7 × 10^5^ normal human keratinocytes were seeded on the top of the dermis mixture and kept submerged in an *in-house* prepared culture medium for 24h, so the cells could form a monolayer. The models were maintained at an air-liquid interface throughout 7 days, changing the media every 2 days, allowing complete epidermis differentiation and stratification (Pennacchi et al., 2015; Pivetta et al., 2019; Tavares Spagolla Napoleão et al., 2020; Devillard and Marquette, 2023; Gluzezak et al., 2024).

### Endothelialized bioprinted human skin model

The endothelialized human dermal models were bioprinted through a pneumatic extrusion technique, and the hydrogel matrix was composed of a solution of 10% (w/v) gelatin, 2% (w/v) alginate and 4% (w/v) fibrinogen, dissolved in DMEM/EGM2 culture medium (50/50). After adding 1.14 × 10^5^ of fibroblasts and 1 × 10^6^ of HUVEC, the bioink solution was homogenized and transferred to a sterile syringe where it was kept at 21 °C to reach the required rheological properties (Devillard and Marquette, 2023). The bioprinting was then performed using the BioAssemblyBot® 400 robotic 6-axis bioprinter (Advanced Solution LifeScience, United States) to print standardized 0.3 cm^3^ bioprinted tissues (1 cm × 1 cm x 3 mm). A 410 µm diameter, 6.35 mm long needle (Nordson EFD, France) was used to bioprint at a set speed of 10 mm/sec. After bioprinting, tissues were consolidated for 90 min at 37 °C, 5% CO_2_ in a solution composed of 3% (w/v) CaCl_2_ (1.02378.0500, Merck, Sigma-Aldrich), 10 U/mL thrombin (t4648-10KU, Merck, Sigma-Aldrich), and 0.4% (w/v) transglutaminase (ACTIVA VM, Ajimoto, Japan), and then incubated at 37 °C, 5% CO_2_ for 7 days in non-treated 6-well plates (2533453, Corning) 3% (w/v) CaCl_2_, 10 U/mL thrombin, and 0.4% (w/v) transglutaminase, and then incubated at 37 °C, 5% CO_2_ for 7 days in non-treated 6-well plates containing inserts (0.4 μm pore membrane; CellQART, 9300412), with medium changes every 2 days (Pourchet et al., 2017; Devillard and Ca, 2021). After this period, 3.7 × 10^5^ keratinocytes were added to the model, and the tissues were cultured at the air-liquid interface with the appropriate culture medium for 7 days, ensuring epidermal stratification and differentiation (Pennacchi et al., 2015; Pivetta et al., 2019; Tavares Spagolla Napoleão et al., 2020).

### Characterization

Tissues were fixed with a 4% (v/v) paraformaldehyde-based solution (P0014, Diapath) for 24 h at 4 °C, during the time points of 1, 5, 7, 12, 14 and 16 days of *in vitro* culture. Then, they were rinsed with PBS (14190, Gibco, France), progressively dehydrated in 30%, 50% and 70% ethanol (v/v) and stored in the last ethanol bath until paraffin embedding. Paraffin-embedded PFA fixed tissues were cut into 5 μm sections. They were dewaxed with two toluene incubations for 10 min and progressively rehydrated with graded ethanol series and finally distilled water. For routine histology, sections were then stained with hematoxylin and eosin using Fast Quick HE staining kit (010263, Diapath) to highlight the cell organization.

For immunohistological analysis, labeling was performed on dewaxed and rehydrated sections, as described above. Sections were labeled with a primary antibody (Collagen I (20111, Novotec, EN4 (ABCAM, ab8087), pan-cytokeratin (PA1-27114, Invitrogen)) and revelation was processed using ImmPRESS® kit (MP-7601 or MP-7602 depending of the primary antibody used, VectorLaboratories). Collagen I staining allowed us to visualize collagen neosynthesis inside the tissues. The presence of endothelization was assessed by incubating the models with anti-endothelium EN4 antibody and the presence of the epidermal layer was confirmed using a mix of pan-cytokeratins 4, 5, 6, 8, 14, and 16. Cell proliferation inside the tissues was estimated from fluorescent imaging following labeling with green calcein-AM (C1430, ThermoFisher Scientific, France). In a dark room, the tissues were rinsed with PBS (14190, Gibco, France) and then incubated for 30 min at 37 °C with calcein-AM (2 μM). The tissues were observed directly using a fluorescence microscope (Nikon Eclipse Ts2R-FL) and the images were captured using a high-definition color DS3-Fi3 camera and NIS-Element software.

### UVA-induced intracellular ROS production

After the reconstructed human skin models and the endothelialized human skin models were fully differentiated on day 10 and 16, respectively, they were placed into new 24 or 6-well plates, and the measurement of intracellular ROS production began incubating them in the dark with DCFH_2_-DA probe (50 μM) (Sigma-Aldrich Brasil Ltda., d6883) for 45 min. After PBS washing, 25 μL of compound Trz-HBH_3_ at 500 ug/mL diluted in sesame oil were applied on top of each skin model for 1 h (Reis et al., 2019; Gluzezak et al., 2024). Then, the tissues were submitted (+UV) or not (-UV) to 10 J/cm^2^ of UVA radiation emitted by a sun simulator (SOL-500 Dr Honle AG, Planegg, Germany) or an UVA lamp (CAMAG, 960914). Immediately after irradiation and washing with PBS, the tissues were frozen in liquid nitrogen and histological sections were obtained in a cryostat. Pictures were taken in an inverted Ti-S microscope (Nikon Instruments Inc., Holland), 488 nm, using 100 m of exposure intensity and analyzed by ImageJ software (Rasmussen et al., 2010; Marionnet et al., 2014; Gluzezak et al., 2024). Results of fluorescence intensity were normalized to area/pixels and expressed as percent fluorescence compared to the non-treated irradiated (NT + UV) control.

## Data Availability

The raw data supporting the conclusions of this article will be made available by the authors, without undue reservation.
